# Is melancholia a distinct syndrome? Recurrence, chronicity, and severity give evidence in the 50 year follow-up of the Lundby Study

**DOI:** 10.3389/fpsyt.2023.1216431

**Published:** 2023-08-04

**Authors:** Linnéa Nöbbelin, Mats Bogren, Cecilia Mattisson, Sofie Westling, Louise Brådvik

**Affiliations:** ^1^Department of Clinical Sciences, Division of Psychiatry, Lund University, Malmö, Sweden; ^2^The Lundby Study, Department of Clinical Sciences, Division of Psychiatry, Lund University, Lund, Sweden

**Keywords:** melancholic depression, depression, course, recurrence, epidemiology, suicide

## Abstract

**Introduction:**

Whether melancholia is a distinct syndrome has long been debated. One aspect of a valid syndrome is whether it allows for determination of a prognosis. The aim of this study is to investigate the course of melancholic depression versus non-melancholic depression with a focus on: (i) time to and probability of recovery from the first depressive episode, (ii) time to and risk of the first recurrence, (iii) rate of recurrence, (iv) time with depression or antidepressant medication, and (v) suicide risk.

**Methods:**

The Lundby Study is a longitudinal community study on mental health that followed a geographically defined population (*N* = 3,563) for up to 50 years, 1947–1997. Subjects with first onset depression were assessed as melancholic (*N* = 46) or non-melancholic (*N* = 381) using the DSM-IV melancholic specifier. These diagnoses were made in retrospect using all available information from semi-structured interviews by psychiatrists, key informants, registers, and patient records.

**Results:**

We found no significant difference between melancholic- and non-melancholic depression in time to and probability of recovery from the first depressive episode. The time to first recurrence was shorter in melancholic than in non-melancholic depression and the risk of first recurrence for the melancholic group was 2.77 (95% confidence interval [CI] 1.83–4.20) times the risk in the non-melancholic group. The median rate of recurrence was higher in the melancholic group, at 0.19 recurrences per year at risk (interquartile range [IQR] 0.08–0.47), compared to the non-melancholic group, at 0.10 recurrences per year at risk (IQR 0.05–0.21) (*p* < 0.03). The median percentage of time being depressed or on antidepressant medication was higher in the melancholic group, 17% (IQR 3–20%), compared to the non-melancholic group, 8% (IQR 7–33%) (*p* < 0.001). The risk of suicide was higher in the melancholic group, hazard ratio 4.13 (95% CI 1.49–11.48, *p* < 0.01).

**Discussion:**

To conclude, melancholic depression had a more recurrent, chronic, and severe course with a higher suicide risk than did non-melancholic depression in the Lundby population. Although our use of retrospective diagnosis might limit interpretation of results, the findings indicate that melancholia may be useful in determining prognosis and may be a valid psychopathological syndrome.

## 1. Introduction

The classification of depressive disorders into valid subtypes reflecting different underlying etiologies has long been debated (1, 2). The current diagnostic classification of depressive disorders (3) has been criticized for arbitrarily grouping depressive states with nonspecific features and for splitting up possibly related disorders into different categories (1, 4, 5). At the beginning of the 20th century, melancholia was viewed as a central and qualitatively distinct depressive disorder associated with psychomotor disturbances, nonreactive mood and a biological etiology rather than the result of negative life events (6). However, research over the course of the century failed to show a clear and reproducible qualitative distinction between melancholic and non-melancholic depression. Thus, melancholia came to be viewed as a severe form of major depression coded through a specifier in the Diagnosis and Statistical Manual of Mental Disorders, 3rd edition (DSM-III) (6). However, in the late 20th and early 21th century, the debate on the status of melancholic depression has returned (2, 5).

One of the most important features of illness classification is whether a prognosis can be determined (7, 8). Robins and Guze (9) proposed five phases of validating a syndrome, of which phase four is follow-up studies. They argued that the validity of a syndrome should be questioned if subjects frequently fall ill with other disorders that can also explain the initial clinical picture during follow-up or if there is a marked difference in outcome between subjects. There are, however, several challenges in determining illness course. One is differentiating when different patterns of symptoms reflect the same underlying disorder and when they should be viewed as co-morbidities (10). It is not uncommon for a person with recurrent depressive episodes to experience episodes that encompass major depressive disorder (MDD), dysthymia and depressive disorder not-otherwise-specified (NOS) (1). Furthermore, some MDD episodes during an illness course might be specified as melancholic or psychotic, whereas others might not (11). Whether all these episodes reflect the same underlying disorder or co-morbid states is unclear. Another challenge when studying disease course are differences in defining outcomes such as recovery and recurrence (12), which can make comparisons between studies difficult.

Melancholic depression has historically been thought to have a more episodic course than other types of depression (13), but previous studies paint a more complex picture. When comparing the length of depressive episodes, some studies have shown that melancholic depressive episodes have shorter durations than do non-melancholic episodes (14, 15). However, other studies have shown no difference (16, 17) and one study showed longer episodes in melancholic depression (18). Regarding risk of recurrence, some studies have found that there is a higher risk in melancholic than in non-melancholic depression (14), while others have found no difference (11, 16, 17, 19). In a previous study (20) in the Lundby population on risk factors for recurrence of depression, severe melancholic depression according to Taylor and Fink (21), was a risk factor for recurrence alongside nervous/tense personality traits and young age at onset. Furthermore, the risk of readmittance to psychiatric inpatient care has been investigated. Two studies (19, 22) showed that subjects with melancholic depression have a higher risk of readmittance than do subjects with other types of depression, whereas one showed no difference in risk (23) and yet showed a higher risk of readmittance for other types of depression (24). Concerning chronicity, one study (11) has shown that subjects with episodes of melancholic depression, compared to subjects with undifferentiated episodes of depression, have a higher percentage of years being symptomatic, a tendency towards higher prescription rates, and a lower rate of stable recovery after the first depressive episode. Depression is an important risk factor for suicide (25). However, the impact of the specific depressive subtype on suicide risk has less often been studied. In one study there was no difference in suicide risk between endogenous, neurotic, and reactive depression (24), whereas another study indicated that subjects with melancholic depression had a higher rate of non-fatal suicidal behavior (17).

Most studies on the course of melancholic depression have been conducted in samples undergoing specialized psychiatric care (14–17, 19, 22–24, 26); far fewer have examined community samples (11). Some studies focus on the current episode while incorporating questions on former episodes (14, 18), while others are longitudinal studies with follow-up times ranging from 6 months (17) to 29 years (24). The diagnostic system most commonly used is the DSM-IV (11, 14–16). Most previous studies (15–18, 23) compared the course between melancholic and non-melancholic depression; however, some studies compared the course of endogenous and neurotic depression (19, 22) and others between melancholic, atypical, and undifferentiated depression (11, 14). Some studies specifically looking at the course of melancholic depression have based their classification of melancholic and non-melancholic on the subtyping of the first-ever depressive episode in an illness course (16, 23). One advantage of this method is that in later episodes, secondary factors such as medication may influence the characteristics of the episode and thus bias the diagnosis. However, in most studies on the illness course, the division into melancholic and non-melancholic subtypes is based on the characteristics of the first depressive episode observed during the study, irrespective of whether it was the first-ever episode or a recurrent one (14, 18, 24). Notably, there are examples in psychiatric diagnostic practice where a later episode defines the disorder, such as a manic episode in bipolar disorder (27).

The Lundby Study is a prospective population-based investigation of mental health that followed all people who resided in two adjoining parishes in the south of Sweden in 1947 or 1957 until 1997 (including those who moved away from the parishes after 1947 and into the parish in 1957; *N* = 3,563, 1823 men,1740 women). Four field investigations have taken place: in 1947, 1957, 1972, and 1997 (28). Data were collected through semi-structured interviews by psychiatrists, speaking with key informants, and examination of registry data and patient records. The attrition rate was between 1 and 2% in the follow-ups in 1957 and 1972, and 6% in 1997 (28). It has been suggested that the best method of studying the natural history of psychopathology (i.e., onset, course, and outcome of a disorder) is a prospective, longitudinal study design with at least two waves of investigation in a community setting and only including subjects with a first lifetime onset of the disorder in the analysis (29). The Lundby Study, which meets all these criteria, offers a unique opportunity to study the course and outcome of melancholic depression.

The aim of this study is to investigate the course and outcomes of melancholic depression compared to non-melancholic depression, defined in accordance with the specifier in the DSM-IV (3). The features examined include (i) time to and probability of recovery from the first-ever depressive episode, (ii) time to and risk of first recurrence, (iiia) rate of recurrence in participants with at least two episodes of depression and (iiib) rate of recurrence after the first recurrence in participants with at least three episodes of depression, (iv) percentage of study time with depression or antidepressant medication, and (v) suicide risk.

## 2. Methods

### 2.1. Diagnostic assessment

At the beginning of the Lundby Study, neither the DSM nor the International Classification of Diseases and Related Health Problems (ICD) were in use. As the clinical classification at that time was deemed unfit for an investigation of a normal population, a diagnostic nomenclature adjusted to community field work was constructed. This Lundby diagnostic system has, with some modifications made in 1957 and 1997, been used throughout the years (28). In 1997, diagnoses in accordance with the DSM-IV (3) and ICD-10 (30) were added. The diagnoses were agreed on by the fieldworkers, all of whom were psychiatrists, using all available information from the semi-structured interviews, key informants, registers, and patient records. The degree of impairment for every episode of psychiatric disorder was rated as mild, medium, severe, or very severe (31). In 1997, the degrees of impairment were approximated to Global Assessment of Functioning (GAF) scores (3), as follows: mild degree of impairment corresponds to GAF 61–70, medium degree corresponds to GAF 51–60, and severe and very severe to GAF 1–50 (32).

### 2.2. Depression in the Lundby Study

The criteria for depression in the Lundby diagnostic system are as follows:

“Lowered mood, depressive feelings, tendency to guilt feelings, gloomy outlook, reduced activity, lack of initiative, reduced self-esteem, lowered enjoyment of life and a feeling of low vitality, anxiety and fear. Has more difficulty than usual, and is often unable to carry out his daily responsibilities. Sometimes retardation is present. The subject is often worse in the morning and better towards the evening. Often, he has sleep disturbances and wakes up in the early morning. Loss of appetite and weight” (33).

The Lundby diagnostic system also includes a diagnosis of “depression plus other psychiatric symptoms” (depression+), which applies to cases with the previously described symptom pattern accompanied by significant other mental symptoms, such as anxiety, obsessive symptoms, and delusions. Sixty percent of the first onset episodes of Lundby depression with medium or worse impairment in 1947–1997 corresponded to the DSM-IV criteria for MDD, whereas the rest corresponded to other DSM-IV mood disorders, predominantly depressive disorder NOS or adjustment disorder with depressed mood (34). The Lundby diagnostic system does not include bipolar disorder. Manic episodes in subjects with what would currently be classified as bipolar disorder were recorded as other psychosis and episodes with depression as depression or depression+ in the Lundby diagnostic system. In the retrospective DSM-IV-based reevaluation of the sample, five subjects with Lundby depression were deemed to meet the DSM-IV criteria for bipolar depression. Lundby depression of mild impairment in most cases does not reach the threshold for a DSM-IV disorder (35) and where excluded from the current study.

### 2.3. Melancholic and non-melancholic depression

We retrospectively assessed all episodes of Lundby depression of “medium” to “very severe” impairment in subjects who had their first lifetime episode during the study as melancholic or non-melancholic in accordance with the DSM-IV melancholic specifier (3). Subjects were considered to have a melancholic-type depressive disorder if they had at least one episode of MDD with melancholic features during the study, irrespective of it being the first-ever depressive episode or a recurrent one. Although bipolar depression has been suggested to be closely related to melancholic depression (36), it is currently (in the DSM-5) viewed as a separate disorder from depressive disorders, acting as a bridge between depressive disorders and the schizophrenia spectrum and other psychotic disorders (37). In the Lundby Study, there are too few subjects with bipolar depression to separately analyze them in comparison to those with MDD with melancholic features. Therefore, all subjects diagnosed with bipolar disorder were excluded from the analysis even if their depressive episodes could be described using the melancholic specifier.

### 2.4. Episode duration

During the field investigations, the month and year of onset of and recovery from the depressive episodes were recorded. If the exact month was difficult to recall, the month of July was recorded. If there were no medical records, the date of onset was based on the subject’s memory of when they first experienced symptoms that developed into a depressive disorder and the date of recovery was based on their memory of when all symptoms were gone and any treatment with antidepressant medication had ended. If medical records and interviews yielded conflicting information about the onset or termination of an episode, the information from the medical records was given precedence as they were documented closer in time to the episode (34). There were no subjects in the current study with depressive episodes occurring less than 2 months apart. Consequently, all subsequent episodes could be classified as recurrences according to the DSM-IV (3).

### 2.5. Suicide risk

Information about suicide cases was retrieved from the Swedish Cause of Death Register. In the current study, the suicide outcome includes both suicides and self-inflicted death by undetermined intent in accordance with different revisions of the ICD. The classification codes for suicide until 1994 were ICD-8 (38)/ICD-9 (39) E950–959 and between 1994 and 1997 ICD-10 (40) X60–84. The classification codes for self-inflicted death by undetermined intent used between 1968, when the concept was first introduced, and 1994 were ICD-8/ICD-9 E980–989 and those between 1994–1997 were ICD-10 Y10–34.

All suicides and cases of self-inflicted death by undetermined intent that occurred during the study period (1947–1997), irrespective of whether they were connected with a depressive episode or not, were included.

### 2.6. Statistical analysis

Subjects were censored from the analyses by dying, withdrawal, study termination or falling ill with “age psychosis,” organic syndrome, schizophrenia or other types of psychoses according to the Lundby diagnostic system (41). In the Lundby diagnostic system these diagnoses were of higher order and excluded the possibility of being diagnosed with depression and depression+. Age psychosis is closely related to dementia, whereas organic syndrome includes cognitive decline due to medical conditions or trauma.

We used Kaplan–Meier analysis to examine differences in time to recovery from onset of the first-ever depressive episode and time between recovery and first recurrence between the melancholic and non-melancholic depression groups. Probability of recovery from the first depressive episode and risk of recurrence were analyzed with Cox regression. In both Cox regression models, the results were adjusted for gender, age and level of impairment at first onset.

When calculating the overall rate of recurrence of depression, we divided the total number of recurrences in subjects who recovered from their first depressive episode by the time in years that participants were free from depressive illness between recurrences (i.e., the time the participants were at risk of recurring). In this analysis, only subjects with at least one recurrence during the study, i.e., at least two episodes of depression, were included. When calculating the rate of subsequent recurrences (following the first recurrence), the number of recurrences excluding the first was divided by the time in years the subjects were free from depressive illness between subsequent recurrent episodes. Only subjects with at least two recurrences were included in this analysis. Differences in the median rates of recurrence and subsequent recurrences between melancholic and non-melancholic depression were analyzed using the Mann–Whitney U test.

We also compared the percentage of time with depression or antidepressant medication between the melancholic and non-melancholic depression groups using the Mann–Whitney U test. To adjust for gender, age at first onset, and impairment of the most severe depressive episode, we used linear regression. In the regression analysis, the outcome variable was logarithmized to obtain a linear relationship between outcome and predictor variables and a model with errors that are more equally distributed around the regression line. In the analyses, subjects who never recovered from the first episode were excluded and studied separately. This was due to difficulties in finding a reasonable model that could fit the distribution pattern when including those subjects who were ill 100% of the time.

Suicide risk was analyzed using Cox regression. The time-to-event was calculated from the onset of the first depressive episode to suicide or censoring. The results were adjusted for gender, age at onset, and impairment of the most severe depressive episode.

In all multivariate models, we tested for inclusion of multiple other variables (i.e., all variables included in Table 1) using forward selection. None resulted in a 10 percent or more change in the estimate (CIE) and did not reach our threshold for confounding (42). The assumption of proportional hazards function over time in the Cox regression models was checked graphically using log–log survival plots. All calculations and analyses were performed with IBM SPSS Statistics 27.

**Table 1:** Sociodemographic, somatic, and psychiatric key-variables by melancholic and nonmelancholic depression.

	Melancholic depression, N 46	Non-melancholic depression, N 381	*p*-value^h^
Mean age at onset (SD)	46.5 (14.4)	47.0 (17.2)	0.85
Gender			0.14
Women	33 (71.7%)	231 (60.6%)	
Men	13 (28.3%)	150 (39.4%)	
Socioeconomic status^a^ at onset (%)			0.04
No information	0 (0.0%)	23 (6.0%)	
White-collar^b^	3 (6.5%)	82 (21.5%)	
Blue-collar^c^	34 (73.9%)	223 (58.5%)	
Self-employed^d^	9 (19.6%)	53 (13.9%)	
Marital status at onset (%)			0.42
Unmarried	9 (19.6%)	118 (31.0%)	
Married/cohabiting	34 (73.9%)	242 (63.5%)	
Divorced	2 (4.3%)	11 (2.9%)	
Widowed	1 (2.2%)	10 (2.6%)	
Nervous tense^e^ before onset (%)			0.07
No	21 (45.7%)	227 (59.6%)	
Yes	25 (54.3%)	154 (40.4%)	
Somatic co-morbidity^f^ before onset (%)			0.78
No	17 (37.0%)	133 (34.9%)	
Yes	29 (63.0%)	248 (65.1%)	
Psychiatric co-morbidity^g^ before onset (%)			0.45
No	31 (67.4%)	235 (61.7%)	
Yes	15 (32.6%)	146 (38.3%)	
Psychiatric co-morbidity^g^ at any time (%)			0.44
No	25 (54.3%)	184 (48.3%)	
Yes	21 (45.7%)	197 (51.7%)	
Impairment at onset			0.008
Medium	29 (63.0%)	305 (80.1%)	
Severe/very severe	17 (37.0%)	66 (19.9%)	
Impairment of most severe episode			<0.001
Medium	19 (41.3%)	291 (76.4%)	
Severe/very severe	27 (58.7%)	90 (23.6%)	

a In accordance with Swedish socioeconomic classification, 1982.

b Assistant and intermediate non-manual employees, employed and self-employed professionals, higher civil servants and executives.

c Unskilled, semiskilled, and skilled manual workers.

d Other than professionals.

e A dichotomous personality factor including traits such as easily feeling uncertain, anxious, insecure, strained, tense and worried.

f Any somatic illness/diagnosis at the field-study closest ahead of first onset of depressive illness.

g A psychiatric disorder in accordance with the Lundby diagnostic groups of Lundby anxiety, tiredness, mixed neurosis and child neurosis. Alcohol use disorder was also taken into account.

h Categorical variables analyzed with Chi-square test of association and continuous variables with independent samples *t*-test.

## 3. Results

### 3.1. Study sample

**Figure 1:**
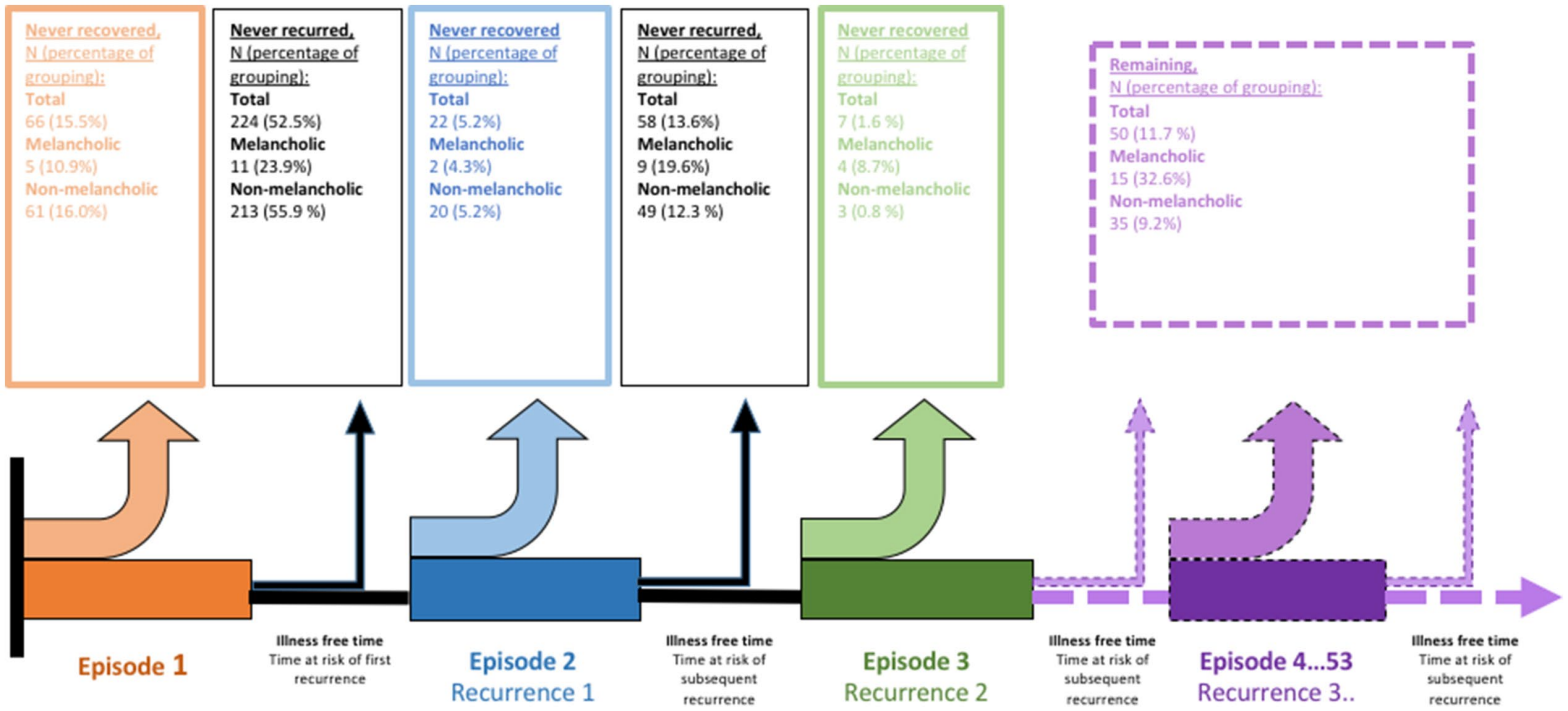
Illustration of the course of depressive illness among subjects in the Lundby study diagnosed with first life-time onset of depression during study inclusion by melancholic and non-melancholic depressive disorder in accordance with DSM-IV.

After excluding subjects diagnosed with bipolar depression (*N* = 5), 427 subjects were diagnosed with a first onset of Lundby depression of medium or worse impairment during the study. Forty-six of these subjects had at least one episode specified as melancholic according to DSM-IV criteria (3) and were assigned to the melancholic depression group. Among the subjects diagnosed with melancholic depression, 34 (73.9%) were specified as melancholic at their first depressive episode, 6 (13.0%) at their second, and 6 (13.0%) at later episodes. The distribution of key sociodemographic, somatic, and psychiatric variables in the melancholic and non-melancholic groups can be seen in Table 1. An illustration of the illness courses for melancholic and non-melancholic depression is shown in Figure 1. As expected, subjects diagnosed with melancholic depression had episodes of more severe impairment than did subjects diagnosed with non-melancholic depression. In the study sample, between 1972 and 1997, 39.3% of the subjects (11 out of 28) with melancholic depression and 14.0% (33 out of 235 subjects) with non-melancholic depression received inpatient care. There were also a difference in socioeconomic status between the groups, in that subjects with melancholic depression more often had blue-collar and less often white-collar professions than did subjects with non-melancholic depression.

### 3.2. Time to and probability of recovery from the first-ever depressive episode

In the melancholic depression group (*N* = 46), five subjects (10.9%) never recovered from their first-ever depressive episode (Figure 1 and Table 2). After falling ill with depression, they were followed for a median of 2 months [interquartile range (IQR) 1.5–93.5]: three (60.0%) died by suicide, one died of other causes, and one had not recovered by the time the Lundby Study terminated in 1997. In the group with non-melancholic depression (*N* = 381), 61 subjects (16%) never recovered (Figure 1 and Table 2). They were followed for a median of 33 months (IQR 14.5–91.5). The most common reason for censoring was study termination (29 subjects, 47.5%) followed by death by other causes (19 participants, 31.1%). Eight (13.1%) died by suicide. The participants who never recovered were followed for less time compared to the rest of the study sample (*p* < 0.001).

**Table 2:** Follow-up time and reasons for censoring by melancholic and non-melancholic depression in the total study sample, the group of cases who never recovered from their first depressive episode and the group of cases who never had a recurrence in depression after recovering from their first depressive episode.

	Never recovered		Never recurred		Total study sample	
	Melancholic depression	Non-melancholic depression	Melancholic depression	Non-melancholic depression	Melancholic depression	Non-melancholic depression
*N* (% of diagnostic group)	5 (10.9%)	61 (16.0%)	11 (26.8%)	213 (66.6%)	46 (100%)	381 (100%)
Median follow-up time, months (IQR)	2 (1.5–93.5)	33 (14.5–91.5)	284 (251.0–339.0)	247 (88.5–332.5)	303.5 (109.8–407.5)	221 (86.0–348.0)
Median follow-up time after first recovery, months (IQR)	.	.	265 (240.0–318.0)	218 (88.5–332.5)	276 (96.8–374.0)	181 (43.5–324.0)
Reason for censoring, *N* (% of column group)						
Lundby Study termination	1 (20.0%)	29 (47.5%)	5 (45.5%)	143 (67.1%)	18 (39.1%)	237 (62.2%)
Withdrawal	0 (0.0%)	1 (1.6%)	0 (0%)	2 (0.9%)	0 (0.0%)	3 (0.8%)
Death other than suicide	1 (20.0%)	19 (31.1%)	5 (45.5%)	51 (23.9%)	16 (34.8%)	96 (25.2%)
Suicide^a^	3 (60.0%)	8 (13.1%)	0 (0.0%)	2 (0.9%)	7 (15.2%)	12 (3.1%)
Organic syndrome or age psychosis^b^	0 (0.0%)	4 (6.6%)	1 (9.1%)	15 (7.0%)	4 (8.7%)	32 (8.4%)
Psychotic disorder^c^	0 (0.0%)	0 (0.0%)	0 (0%)	0 (0%)	1 (2.2%)	1 (0.3%)

IQR, inter quartile range.

a Both suicides (17 subjects) and self-inflicted death by undetermined intent (2 subjects).

b There were only 2 subjects who were censored due to falling ill in organic syndrome.

c Both subjects fell ill in psychotic disorder UNS.

In both melancholic and non-melancholic groups, it took approximately 11 months for 50% of the group to recover from the first-ever depressive episode. After adjusting for gender, age, and impairment at onset, there was no significant difference between the groups in probability of recovery from the first depressive episode, hazard ratio (HR) 1.34 (95% CI 0.96–1.86) (Table 3A).

**Table 3:** Results from analyses of course variables by melancholic and non-melancholic depression in accordance with DSM-IV melancholic specifier.

(A) Recovery after first depressive episode								
	N	Time to recovery from first onset (months)^a^			Cox regression on time to recovery^b^			
		Median	95% CI		HR	95% CI		*p*-value
			LB	UB		LB	UB	
Melancholic depression	46	11	6.74	12.26	1.34	0.96	1.86	0.09
Non-melancholic depression	381	11	9.65	12.35	1			

(B) First recurrence in depression								
	N	Time to first recurrence from recovery (months)^a^			Cox regression on time to first recurrence^b^			
		Median	95% CI		HR	95% CI		*p*-value
			LB	UB		LB	UB	
Melancholic depression	41	67	0.00	155.19	2.77	1.83	4.20	<0.001
Non-melancholic depression	320	475	342.02	607.98	1			

(C) Rates of recurrence in depression								
	Rate of recurrence (number of recurrences per year at risk)^c^				Rate of subsequent recurrence (number of subsequent recurrences per year at risk)^d^			
	N	Median	IQR	*p*-value^e^	N	Median	IQR	*p*-value^e^
Melancholic depression	30	0.19	0.08–0.47	0.03	19	0.39	0.15–0.92	0.14
Non-melancholic depression	107	0.10	0.05–0.21		38	0.16	0.08–0.97	

(D) Percentage of study time being depressed or on antidepressant medication								
	N	Median percentage			Linear regression on log percentage of time being depressed or on antidepressant medication^f^			
		Median	IQR	*p*-value^e^	B	95% CI		Value of *p*
						LB	UB	
Melancholic depression	41	0.17	0.07–0.33	<0.001	0.24	0.06	0.43	0.01
Non-melancholic depression	320	0.08	0.03–0.20					

(E) Suicide risk^g^					
	N	HR	95% CI		*p*-value
			LB	UB	
Melancholic depression	46	4.13	1.49	11.48	<0.01
Non-melancholic depression	381	1			

*N*, number of cases in the analysis; CI, confidence interval; LB, lower bound; UB, upper bound; HR, hazard ratio; IQR, inter quartile range; B, regression coefficient.

a Kaplan Meier survival analysis was used in the calculations.

b Multivariate cox-regression models adjusted for age at onset, gender, impairment at onset. The hazard ratios were proportional over time as assessed graphically using log–log survival plots.

c Number of recurrences divided by time in years free from depressive illness since recovery from first onset. Only cases with at least one recurrence were included.

d Number of recurrences excluding the first recurrence divided by the time in years free from depressive illness since recovery from the first recurrence. Only cases with at least 2 recurrences were included.

e Mann–Whitney U test.

f Multivariate linear regression adjusted for age at onset, gender and impairment of most severe episode. The outcome/dependent variable is log percentage of time being depressed or on antidepressant medication. Cases who never recovered were excluded from the analysis.

g Multivariate cox-regression model adjusted for age at onset, gender, impairment of most severe episode. The hazard ratios were proportional over time as assessed graphically using log–log survival plots.

### 3.3. Time to and risk of recurrence of depression

In the melancholic group (*N* = 46), 11 subjects (26.8%) never had a recurrence after recovering from their first-ever depressive episode (i.e., stable recovery), whereas in the non-melancholic group (*N* = 381), 213 subjects (66.6%) showed stable recovery (Figure 1 and Table 2). The estimated time it took for 50% of the group to have their first recurrence was 67 months (95% CI 0.00–155.19) in the melancholic group and 475 months (95% CI 342.02–607.98) in the non-melancholic group. The melancholic group had 2.77 (95% CI 1.83–420, *p* < 0.001) times the risk of first recurrence of the non-melancholic group, after adjusting for gender, age, and impairment at onset (Table 3B).

The median overall rate of recurrence in the melancholic group was 0.19 per year at risk, which was significantly higher than the rate of recurrence in the non-melancholic group, 0.10 per year at risk, (*p* = 0.03) (Table 3C). The median rate of subsequent recurrence was 0.39 per year at risk in the melancholic group compared to 0.16 in the non-melancholic group. However, this difference was not statistically significant (Table 3C).

### 3.4. Time with depression or treatment with antidepressant medication

The median percentage of study time showing symptoms of depression or being treated with antidepressant medication was higher in the melancholic group than in the non-melancholic group, at 17% (IQR 7–33%) vs. 8% (IQR 3–20%), respectively (*p* < 0.001). The difference remained significant after adjusting for gender, age at onset, and impairment of the most severe episode (Table 3D).

### 3.5. Suicide risk

After adjusting for gender, age at onset, and impairment of the most severe episode, the suicide risk during the study period in the melancholic group was 4.13 (95% CI 1.49–11.48, *p* < 0.01) times that of the non-melancholic group (Table 3E).

## 4. Discussion

### 4.1. Main findings

The results indicate that melancholic depression has a more recurrent, chronic, and severe course than does non-melancholic depression. This was reflected in how the melancholic group had a higher risk of first recurrence and overall rate of recurrence, more episodes of greater severity, a lower proportion of participants with stable recovery, a higher percentage of time being depressed or on antidepressants, and a greater suicide risk. The rate of subsequent recurrence (i.e., recurrences following after the first recurrence) among subjects with at least two recurrent episodes was also higher in the melancholic group than in the non-melancholic group, albeit not significantly. The non-significant result may have been due to the small sample size.

There was no difference between subjects with melancholic and non-melancholic depression in time to recovery after onset of the first depressive episode. The results concerning episode length between melancholic and non-melancholic depression are very mixed in the literature (14–16, 18), indicating that there is no consistent difference.

Most previous studies comparing the course of melancholic and non-melancholic depression found no difference in the risk or rate of recurrence between these groups (11, 16, 17, 19). This may be due to methodological differences between these studies and the current one. Three of the previous studies were conducted in specialized care settings (16, 17, 19), which likely reduced differences between the melancholic and non-melancholic diagnostic groups due to sampling bias. Further, two of the previous studies followed their samples for considerably less time than did the current study, at 6 months (17) and 18 months (16). In the current study, the median time to the first recurrence for the melancholic group was 67 months (95% CI 0.00–155.19) and that for the non-melancholic was 475 months. Consequently, recurrences might go unrecorded in studies with follow-up periods of shorter duration. In the community-based Zurich Study (11), a group of 192 subjects diagnosed with DSM-IV MDD was followed for 21 years. These subjects were divided into four, mutually exclusive, groups based on DSM-IV subtypes: (i) melancholic and atypical episodes, (ii) melancholic episodes, (iii) atypical episodes, and (iv) unspecified depressive episodes only. Illness course was divided into single episode, recurrent, and chronic. A chronic course was defined as being depressed for at least 50% of the days during 1 year. These outcomes are not fully in line with the ones in our study. However, the single episode group in the Zurich Study overlaps with the group in the current study who did not ever show recurrence after recovering from the first depressive. In the two groups with melancholic depressive episodes in the Zurich Study, 4 and 16% had only a single episode, whereas in the group with unspecified depressive episodes only, this proportion was 40%. This difference, although not significant in the Zurich Study, is similar to the difference in the current study between the melancholic and non-melancholic groups in which 26.8 and 66.6%, respectively, never recurred after recovering from the first depressive episode.

Post (43) proposed that with each episode of depression in a recurrent depressive illness, the episodes become longer and more frequent and the course becomes more self-acting through a sensitization process. Accordingly, we might hypothesize that the difference in risk of recurrence between melancholic and non-melancholic groups would become increasingly less evident in subjects with increasingly more episodes of depression. However, in the present study, the rate of overall recurrence in subjects with at least two episodes of depression and the rate of subsequent recurrences in subjects with at least three episodes of depression was higher in the melancholic group than in the non-melancholic group. Furthermore, the difference between rates of subsequent recurrences in the melancholic and non-melancholic depression groups was greater than the difference between rate of overall recurrences in the same groups. However, the difference in rates of recurrences between the melancholic and non-melancholic depression groups was not statistically significant, probably because of the small sample size. Nevertheless, these results suggest that the increased risk of recurrence in melancholic compared to non-melancholic depression may hold true throughout the illness course.

In the present study, the percentage of study time being depressed or on antidepressant medication was higher in the group with melancholic depression than in the group with non-melancholic depression. This is in line with the results from the Zurich Study (11), where in both groups with melancholic episodes had higher percentages of years with symptoms of depression and years treated with medication than the group with episodes of unspecified depression only.

In our study, the suicide risk in the melancholic depression group was higher than that in the non-melancholic group. One study has shown no difference in risk of completed suicides between melancholic and non-melancholic depression (24). However, some studies have shown higher rates of suicidal ideation in melancholic depression compared to non-melancholic depression (17, 44, 45). The presence of melancholia may add power to the prediction of suicide risk.

### 4.2. Strengths and limitations

The Lundby Study is a unique prospective community study of a geographically defined complete population suited for studying the course of psychiatric illness. The long follow-up period (up to 50 years) increases the proportion of study subjects who pass through the risk period for depression during the study. Additionally, the integration of information from several sources further increases case finding rates. Care-seeking bias is avoided in a community-study and the low attrition in the Lundby Study further reduces selection bias. However, there are some limitations and methodological aspects to consider.

Diagnosing melancholic depression in retrospect poses some challenges. The Lundby Study stretched over 50 years, during which the diagnostic view on psychiatric illness changed. This might have influenced what kind of information was gathered throughout the study. However, the concept of melancholia has a long history (46) and its features were relatively well known throughout the 20th century (6), being frequently described in case records. Several different diagnostic constructs of melancholic depression apart from the specifiers in the different editions of the DSM have been developed, e.g. the Core system (47) the Sydney Melancholia Prototype Index (the SMPI) (18) and Taylor & Finks concept of melancholia (36). The melancholic specifiers in the DSM editions have been critiqued of simply identifying more severe cases of MDD and not a qualitatively distinct syndrome (48). However, the melancholic specifier in the DSM-IV is still the most widely used diagnostic construct when studying the long-term course of subtypes of depression. Furthermore, in the current study all multivariate analyses adjust for the severity of the depression limiting the possibility of the differences in illness course are simply reflecting a difference in severity between melancholic and non-melancholic depression. Nonetheless, it would have been interesting to study and compare the course of different constructs of melancholia but the Lundby Study is not suited for such a study. During the Lundby Study, data on hormonal-and genetic markers were not gathered and there is no possibility of gathering these in retrospect. As a result, proposed diagnostic constructs including hormonal markers, e.g. Taylor and Finks concept of melancholia, cannot be used. When diagnosing in retrospect, psychomotor disturbances cannot be visually graded and you must rely on reports from the subjects, key informants or case records. Constructs dependent on visually grading psychomotor disturbances, e.g., the Core system, cannot be used. Furthermore, there is no possibility of gathering more information in threshold cases when diagnosing in retrospect. As a result, melancholic depression might to some extent have been underdiagnosed and misclassified as non-melancholic. However, as the clinical presentation of melancholic depression is often more distinct than is other types of depression, the risk of underdiagnosing might be lower.

The incidence of bipolar depression in the Lundby Study, 0.04 per 1,000 person years under risk (49), is lower than in other epidemiological studies (50). One reason for the lower incidence might be that most other large studies uses laymen and structured diagnostic interviews when gathering data whereas in the Lundby Study trained psychiatrists conducted semi-structured interviews probably limiting overinclusion in some disorders. Another possible reasons for the low incidence of bipolar disorder in the Lundby Study is that the diagnosis of bipolar disorder type II was not as widely known during the beginning of the 20th century and first was added as a disorder in the DSM-IV (3). Consequently, the features of hypo mania might not have been reliably recorded in earlier field studies and the cases of bipolar disorder in the Lundby Study are best classified as bipolar disorder type I. Thus, some cases included in the current study might be cases of bipolar disorder type II and not true cases of unipolar depression. As bipolar depression is associated with a more recurrent course (51) this might bias the results. Some studies have suggested an association between bipolar disorder type II and episodes of atypical depression (52) whereas other researchers have seen an association between bipolar disorder broadly defined, but especially bipolar disorder type I, and melancholic depression (21). In conclusion it is difficult to know in what group, the melancholic or non-melancholic depression group, most of the potential cases of bipolar disorder type II would have been wrongly added.

The long interval between field studies in the Lundby Study might also introduce a recall bias, reducing the number of recurrences registered and affecting the ability to remember the date of onset and recovery from a depressive episode. As inpatient care and patient records were more frequent in the melancholic group and the depressive episode more severe, recall bias might have been better compensated in this group than in the non-melancholic group, thus explaining the higher number of recurrences. However, the effects of recall bias were also limited by using other information sources such as key informants, registers and patient records from primary care.

The inclusion of time on antidepressant medication as part of episode duration probably increased the episode duration. As melancholic depression tends to lead to more severe impairment and use of specialized care (15) compared to non-melancholic depression, it might more frequently be treated with antidepressant medication compared to non-melancholic depression. Therefore, the inclusion of time with treatment in the current study might have led to an upward bias in the length of the depressive episodes in the melancholic group, resulting in the lack of a difference in length of the first depressive episodes between the two diagnostic groups. However, if time on medication had not been considered, it would instead have introduced a treatment bias.

In the current study, all Lundby depressive episodes with medium to very severe impairment were included. As a result, a wider range of DSM-IV disorders milder as well as more severe: MDD, depressive disorder NOS and adjustment disorder with depressed mood, were included compared to other studies, which only included episodes of MDD (11, 14, 16). This might have resulted in the greater observed difference in the risk of recurrence between the diagnostic groups, as subjects with a single episode of a milder and not as recurrent depressive disorder might more often have been included in the non-melancholic group. However, the occurrence of different depressive types, milder as well as more severe, during the course of depressive disorder in the same individual is quite common (1) and it remains a matter of debate whether these should be treated as co-morbidities or different expressions of the same underlying disorder. The diagnosis of depressive disorder NOS has been suggested to include prodromes or less severe cases of MDD (53) and to have a similar etiology to MDD (1). There is also evidence of depressive disorder NOS leading to substantial loss of function (1) and the exclusion of these episodes when analyzing the course might not represent the true trajectory of the depressive illness in an individual.

## 5. Conclusion

DSM-IV melancholic depression has a significantly more recurrent, chronic, and severe long-term course than does non-melancholic depression. This is reflected in a higher risk of first recurrence, a lower proportion of subjects with stable recovery, a higher overall rate of recurrence, more episodes, a higher percentage of study time being depressed or being treated with antidepressant medication, and a greater suicide risk in melancholic than in non-melancholic depression. Although there are many methodological challenges, these results suggest that by viewing melancholia as a higher-order depressive disorder, decisive of the diagnosis of the illness, it becomes easier to determine the prognosis. Furthermore, the results stress the importance of a lengthier follow-up and adequate long-term treatment of individuals with melancholic depression and suggest that the syndrome of melancholia is worth considering in the assessment of suicide risk. Although, a distinct course is only one aspect of validating a disorder the results support the idea of melancholia being a distinct syndrome.

## Data availability statement

The raw data supporting the conclusions of this article will be made available by the authors, without undue reservation.

## Ethics statement

The Lundby field-study of 1997 and further use of the already gathered material was approved by the Research Ethics Committee of the medical Faculty at the University of Lund and written consent was attained from all living subjects at that time. However, previous field-studies (1947, 1957 and 1972) predated the current ethical system and written consent was not gathered during these field-studies.

## Author contributions

LN played a role in the study design, analyzed, interpreted and structured the data, and wrote the paper. LB initiated the study, played a role in the study design, revised the paper, and contributed to the interpretation of the data. SW was the main supervisor of the project, revised the paper, and contributed to the interpretation of the data. MB assessed in retrospect the cases of first-onset melancholia of severe impairment, and was one of the main field workers in the 1997 Lundby Study follow-up. He also contributed to the interpretation of the data and revision of the paper. CM was one of the main field workers in the 1997 Lundby Study follow-up and has contributed to the interpretation of the data. All authors contributed to the article and approved the submitted version.

## Funding

Funding for this study was granted from The Ellen and Henrik Sjöbring memorial fund, Lindhaga foundation, Bror Gadelius memorial fund, OM Perssons foundation and the sector for psychiatry and habilitation at Region Skåne. The funding sources had no role in either the study design, the collection, analysis, interpretation of data or in the writing of the paper. Furthermore, they had no role in the decision to submit the paper for publication.

## Conflict of interest

MB has received lecturing and advisory board honoraria from Otsuka, Recordati and the Lundbeck Institute.

The remaining authors declare that the research was conducted in the absence of any commercial or financial relationships that could be construed as a potential conflict of interest.

## Publisher’s note

All claims expressed in this article are solely those of the authors and do not necessarily represent those of their affiliated organizations, or those of the publisher, the editors and the reviewers. Any product that may be evaluated in this article, or claim that may be made by its manufacturer, is not guaranteed or endorsed by the publisher.
